# Supplementary material for: Identification of a New Infectious Pancreatic Necrosis Virus (IPNV) Variant in Atlantic Salmon (Salmo salar L.) that can Cause High Mortality Even in Genetically Resistant Fish
Source: Front Genet. 2021 Nov 26;12:635185. doi: 10.3389/fgene.2021.635185 (PMC8663487; doi:10.3389/fgene.2021.635185)
Supplement: Supplementary file 2 [file Table1.pdf]

Supplementary Table 1. Overview of the laboratory results, confirming the initial diagnosis of the IPNV infection and QTL status of the fish. The extracted RNA from all six head-kidney samples (BG_1 – BG_6) were used for transcriptome sequencing, viral genome detection and subsequent assembly.

Num.	tissue	QTL	qRT-PCR Ct value	histology	Immunohistochemistry
1	head-kidney	Yes	20.32	Not tested	Not tested
2	head-kidney	Yes	19.79	Not tested	Not tested
3	head-kidney	Yes	18.93	Not tested	Not tested
4	head-kidney	Yes	18.92	Not tested	Not tested
5	head-kidney	Yes	20.77	Not tested	Not tested
6	head-kidney	Yes	19.32	Not tested	Not tested
7	adipose fin	Yes	Not tested	Not tested	Not tested
8	adipose fin	Yes	Not tested	Not tested	Not tested
9	adipose fin	Yes	Not tested	Not tested	Not tested
10	adipose fin	Yes	Not tested	Not tested	Not tested
11	adipose fin	Yes	Not tested	Not tested	Not tested
12	adipose fin	Yes	Not tested	Not tested	Not tested
13	adipose fin	Yes	Not tested	Not tested	Not tested
14	adipose fin	Yes	Not tested	Not tested	Not tested
15	adipose fin	Yes	Not tested	Not tested	Not tested
16	adipose fin	Yes	Not tested	Not tested	Not tested
17	multiple	Not tested	Not tested	Multifocal necrosis in the exocrine pancreas	positive
18	multiple	Not tested	Not tested	Multifocal necrosis in exocrine pancreas and multifocal bleeding in abdominal fat	positive
19	multiple	Not tested	Not tested	Multifocal necrosis in exocrine pancreas and focal, extensive necrosis in the liver	positive
