# Supplementary material for: Identification of a New Infectious Pancreatic Necrosis Virus (IPNV) Variant in Atlantic Salmon (Salmo salar L.) that can Cause High Mortality Even in Genetically Resistant Fish
Source: Front Genet. 2021 Nov 26;12:635185. doi: 10.3389/fgene.2021.635185 (PMC8663487; doi:10.3389/fgene.2021.635185)
Supplement: Supplementary file 3 [file Table2.pdf]

Supplementary Table 2. Estimates of evolutionary divergence rate between the amino acid (A) and nucleotide (B) sequences of the polyprotein of the assembled genomes. The rate variation among sites was modelled with a gamma distribution (shape parameter = 1). The actual number of differences are provided in the parentheses.

A	**BG_1_poly**	**BG_2_poly**	**BG_3_poly**	**BG_4_poly**	**BG_5_poly**	**BG_6_poly**
**BG_1_poly**	0 (0)	0 (0)	0 (0)	0 (0)	0.00206 (2)	0.00206 (2)
**BG_2_poly**		0 (0)	0 (0)	0 (0)	0.00206 (2)	0.00206 (2)
**BG_3_poly**			0 (0)	0 (0)	0.00206 (2)	0.00206 (2)
**BG_4_poly**				0 (0)	0.00206 (2)	0.00206 (2)
**BG_5_poly**					0 (0)	0 (0)
**BG_6_poly**						0 (0)

B	**BG_1_poly**	**BG_2_poly**	**BG_3_poly**	**BG_4_poly**	**BG_5_poly**	**BG_6_poly**
**BG_1_poly**	0 (0)	0 (0)	0 (0)	0 (0)	0.00308 (9)	0.00308 (9)
**BG_2_poly**		0 (0)	0 (0)	0 (0)	0.00308 (9)	0.00308 (9)
**BG_3_poly**			0 (0)	0 (0)	0.00308 (9)	0.00308 (9)
**BG_4_poly**				0 (0)	0.00308 (9)	0.00308 (9)
**BG_5_poly**					0 (0)	0 (0)
**BG_6_poly**						0 (0)
