# Supplementary material for: Identification of a New Infectious Pancreatic Necrosis Virus (IPNV) Variant in Atlantic Salmon (Salmo salar L.) that can Cause High Mortality Even in Genetically Resistant Fish
Source: Front Genet. 2021 Nov 26;12:635185. doi: 10.3389/fgene.2021.635185 (PMC8663487; doi:10.3389/fgene.2021.635185)
Supplement: Supplementary file 4 [file Image2.pdf]

Supplementary Figure 2. Neighbour-Joining trees, showing the phylogenetic relationships between the assembled polyprotein (A) and VP1 (B) segments of the IPNV genomes for the six samples sequenced in this study and various strains, with complete sequences, obtained from the NCBI. The trees are constructed based on the amino acid sequence information. The confidence of association between sequences was estimated using bootstrap testing (1000 replicates) and is shown next to the branches. The evolutionary distances were computed using the Poisson correction method (Zuckerkandl and Pauling, 1965) and are in the units of the number of amino acid substitutions per site. The rate variation among sites was modelled with a gamma distribution (shape parameter = 1). All ambiguous positions were removed in a pairwise deletion fashion.

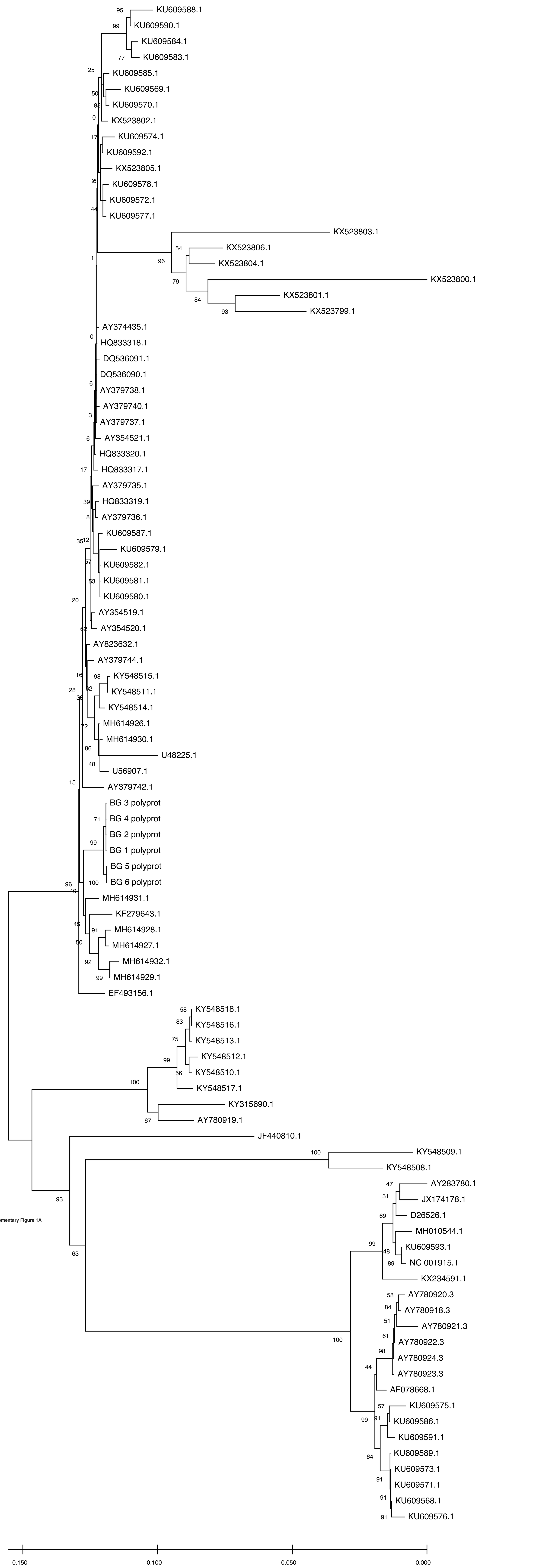

Supplementary Figure 1A

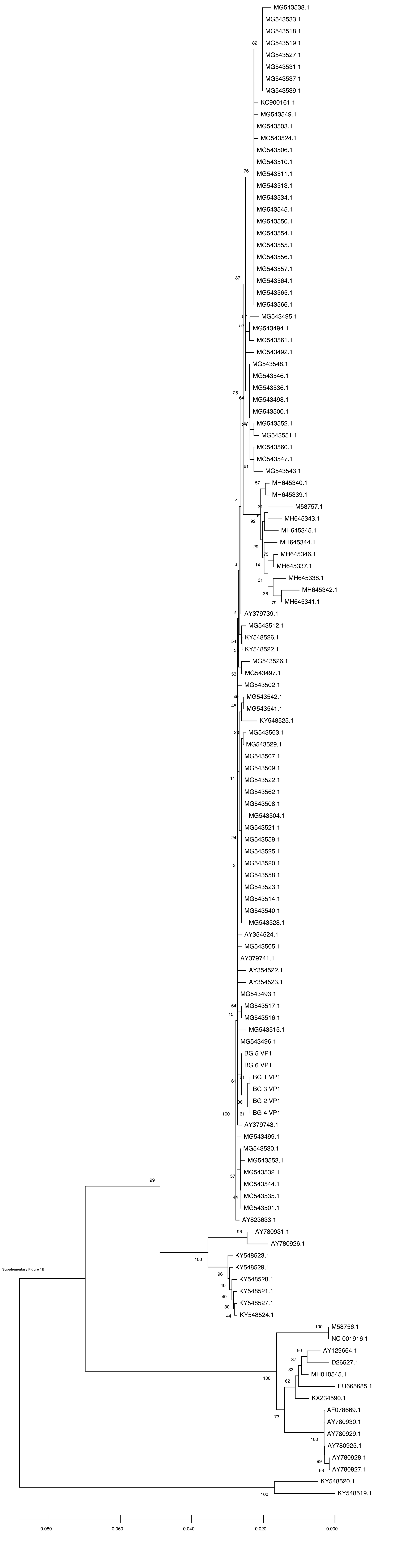
Supplementary Figure 1B
MG543538.1
MG543533.1
MG543518.1
MG543519.1
MG543527.1
MG543531.1
MG543537.1
MG543539.1
KC900161.1
MG543549.1
MG543503.1
MG543524.1
MG543506.1
MG543510.1
MG543511.1
MG543513.1
MG543534.1
MG543545.1
MG543550.1
MG543554.1
MG543555.1
MG543556.1
MG543557.1
MG543564.1
MG543565.1
MG543566.1
MG543495.1
MG543494.1
MG543561.1
MG543492.1
MG543548.1
MG543546.1
MG543536.1
MG543498.1
MG543500.1
MG543552.1
MG543551.1
MG543560.1
MG543547.1
MG543543.1
MH645340.1
MH645339.1
M58757.1
MH645343.1
MH645345.1
MH645344.1
MH645346.1
MH645337.1
MH645338.1
MH645342.1
MH645341.1
AY379739.1
MG543512.1
KY548526.1
KY548522.1
MG543526.1
MG543497.1
MG543502.1
MG543542.1
MG543541.1
KY548525.1
MG543563.1
MG543529.1
MG543507.1
MG543509.1
MG543522.1
MG543562.1
MG543508.1
MG543504.1
MG543521.1
MG543559.1
MG543525.1
MG543520.1
MG543558.1
MG543523.1
MG543514.1
MG543540.1
MG543528.1
AY354524.1
MG543505.1
AY379741.1
AY354522.1
AY354523.1
MG543493.1
MG543517.1
MG543516.1
MG543515.1
MG543496.1
BG 5 VP1
BG 6 VP1
BG 1 VP1
BG 3 VP1
BG 2 VP1
BG 4 VP1
AY379743.1
MG543499.1
MG543530.1
MG543553.1
MG543532.1
MG543544.1
MG543535.1
MG543501.1
AY823633.1
AY780931.1
AY780926.1
KY548523.1
KY548529.1
KY548528.1
KY548521.1
KY548527.1
KY548524.1
M58756.1
NC 001916.1
AY129664.1
D26527.1
MH010545.1
EU665685.1
KX234590.1
AF078669.1
AY780930.1
AY780929.1
AY780925.1
AY780928.1
AY780927.1
KY548520.1
KY548519.1
0.080
0.060
0.040
0.020
0.000
