# Supplementary material for: Identification of a New Infectious Pancreatic Necrosis Virus (IPNV) Variant in Atlantic Salmon (Salmo salar L.) that can Cause High Mortality Even in Genetically Resistant Fish
Source: Front Genet. 2021 Nov 26;12:635185. doi: 10.3389/fgene.2021.635185 (PMC8663487; doi:10.3389/fgene.2021.635185)

Supplementary Figure 1. Neighbour-Joining trees, showing the phylogenetic relationships between partial polyprotein nucleotide (A) and the amino acid (B) sequences of the reported assemblies with an isolate (MH562009) previously recovered from a field outbreak (Kristoffersen et al., 2018). BG 1 to 6 represent assembled sequences in the current study. The distances were computed using the Maximum Composite Likelihood method for nucleotides and Poisson for the amino acid data. The rate variation among sites was modelled with a gamma distribution (shape parameter = 1). There were a total of 1513 nucleotides and 504 amino acids in the final dataset. Evolutionary analyses were conducted in MEGA X.

Supplementary Figure 1A

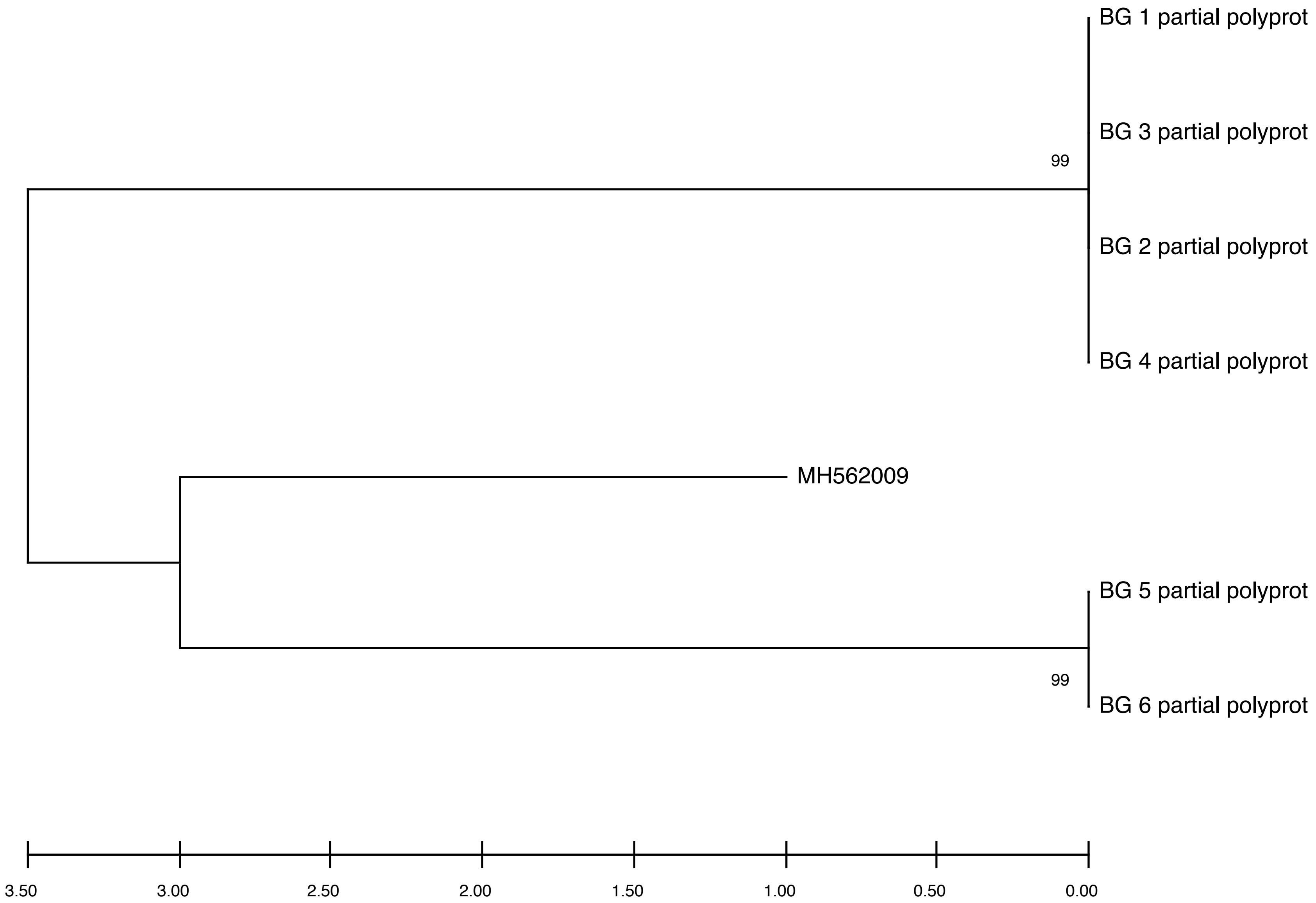

Supplementary Figure 1B

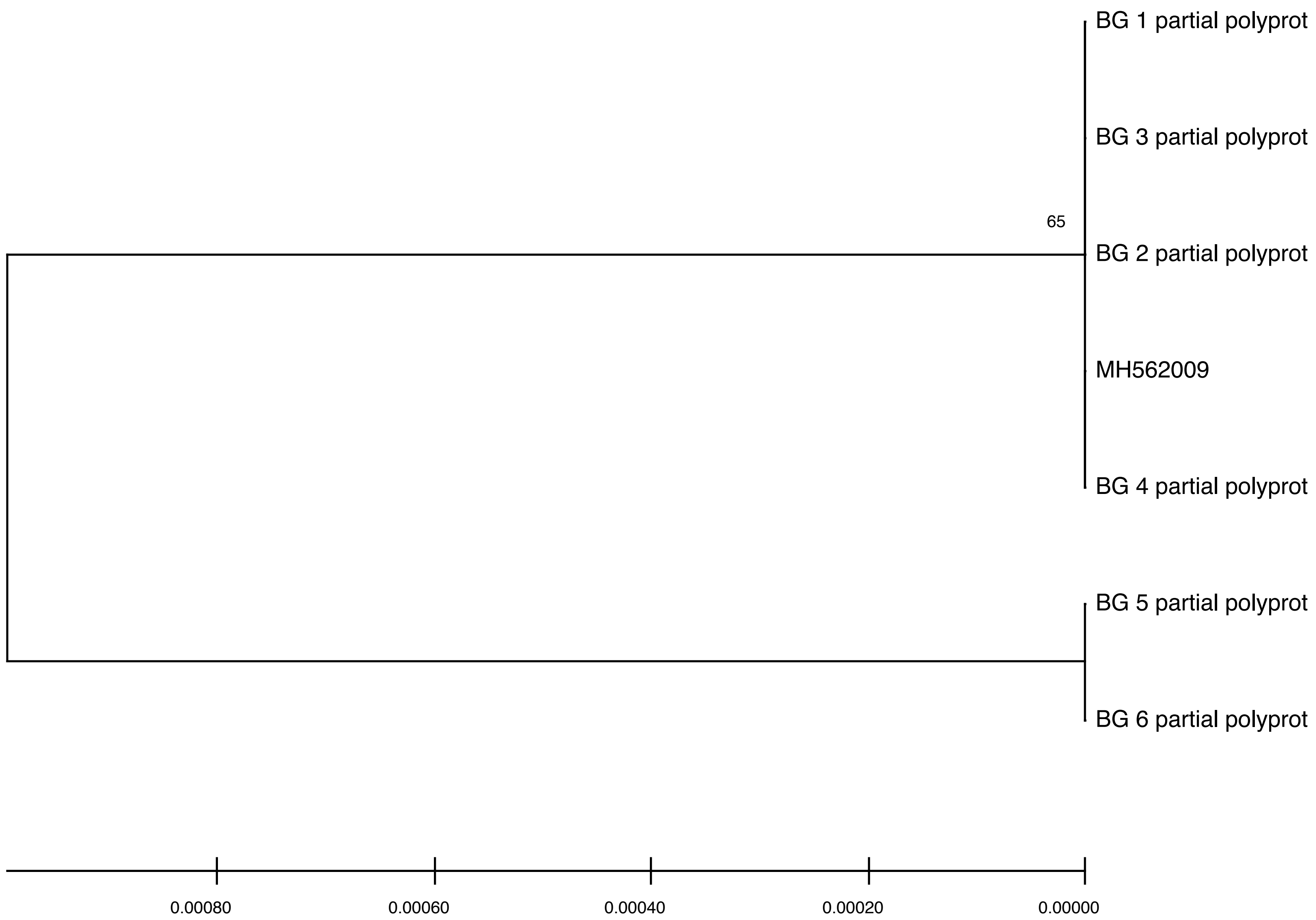

Supplement: Supplementary file 8 [file Image1.pdf]
